# The associations between autistic characteristics and microtransaction spending

**DOI:** 10.1038/s41598-024-64812-z

**Published:** 2024-06-18

**Authors:** Tegan Charnock, Aaron Drummond, Lauren C. Hall, James D. Sauer

**Affiliations:** 1 School of Psychological Sciences, University of Tasmania, Launceston, Australia; 2 School of Psychological Sciences, University of Tasmania, Hobart, Australia; 3 Australian Institute of Health Service Management, University of Tasmania, Hobart, Australia

**Keywords:** Psychology, Human behaviour

## Abstract

Microtransactions provide optional, virtual, video game goods that, for an additional cost to the player, provide additional game content and alter the gameplay experience. Loot boxes—a specific form of microtransaction—offer randomised rewards in exchange for payment, and are argued to be structurally and psychologically similar to gambling. Nascent research suggests that a link exists between autism and both problematic gaming and problematic gambling. Here, we investigated the relationships between autistic characteristics and experiences, and excessive video gaming and microtransaction expenditure. A sample of 1178 adults from Australia, Aotearoa, and The United States were recruited from Prolific Academic, and completed a survey measuring in-game expenditure, autistic characteristics and experiences, problematic gaming, problematic gambling, and risky loot box use. Analyses showed positive associations between autistic characteristics and experiences with problematic gaming and problem gambling symptomatology. However, results also showed a small, negative association between autistic characteristics and experiences and spending on loot boxes when problem gambling symptoms, problematic gaming, and risky loot box use were statistically controlled for. These results suggest that autistic gamers may be vulnerable to problematic gaming and gambling, but that this effect does not extend to the purchasing of microtransactions.

## Introduction

Video gaming is a lucrative industry. In 2022, an estimated 2.46 billion people worldwide played video games, contributing to a global market generating $347USD billion in revenue^1,2^. The ‘microtransaction’ monetisation model has contributed significantly to the economic growth of the industry. Microtransactions are additional in-game purchases made with real world money, and offer users additional virtual in-game items. Microtransactions are characterised by their small financial cost per transaction and typically provide access to small amounts of additional game content. This content allows gamers to augment their gaming experience, typically providing extra game or avatar customisation options, in-game advantages, or extra playable content^3^. Some microtransactions involve purchasing pre-specified content, whereby players know exactly what item they will receive (i.e., a non-randomised microtransaction). In other cases, game users may purchase a ‘loot box’. A loot box is a digital container of randomised virtual items, often purchased with either real world money or with virtual currency. The contents of a loot box are unknown, and provide gamers the chance to acquire rare or helpful in-game items (i.e., a randomised microtransaction)^3^. Microtransaction monetisation models have been very successful. For example, Fortnite—a free-to-play online multiplayer game launched in July 2017—generated $US9.1 billion in revenue for games companies across 2018 and 2019 through microtransactions^4^. However, these models have attracted criticism for being financially exploitative^5^, and in the case of loot boxes, akin to gambling^6^. Here we investigated whether some individuals, such as individuals with neurodiverse characteristics, might be vulnerable to overspending on microtransactions generally, and loot boxes in particular.

Microtransactions have generated significant academic concern. Drawing upon elements of law and behavioural economics, King and Delfabbro^5^ argue that some in-game monetisation models could be considered predatory, whereby in-game purchasing systems are designed to conceal the actual cost of microtransaction expenditure until players are psychologically and financially invested in the game. In these cases, it is theorised that sophisticated video game algorithms and in-game features might encourage microtransaction expenditure^5^. For example, player data (e.g., playing and spending history) may encourage spending by providing tailor-made offers to the individual user, and through games sending unsolicited notifications for ‘limited time’ or special offers^5^. Some games may also present overly complicated purchasing environments that obscure the true cost of microtransaction expenditure—with unintuitive design interfaces, or by requiring users to purchase an intermediate in-game currency that is then used to obtain the desired in-game items^7,8^.

Furthermore, the utilization of loot boxes has been of particular academic concern**.** The mechanisms which underpin many loot box systems share numerous psychological, structural, and legal similarities to conventional forms of gambling^6^. For example, like many gambling activities, loot boxes are purchased with real currency, have randomly determined outcomes, and low probabilities of obtaining a high-value item/s^6^. While gambling activities may yield rewards with a more tangible use (e.g., money), many items won from loot boxes have ostensibly very little or no real-world value. However, high value loot box rewards can potentially provide gamers with substantial in-game advantages (e.g., powerful weapons or protective equipment) that can be used to defeat other players and progress in-game objectives, thus being considered valuable within the gaming context^3^. Further, in some cases, in-game rewards can be on-sold for real world money^6,9^. Both gambling and loot boxes provide rewards on variable ratio reinforcement schedules, whereby positive reinforcers (wins/desired loot box rewards) are provided intermittently to the user according to their behaviour (bets/loot box purchases)^6^. Furthermore, there is a consistent positive association between loot box spending and problem gambling symptomatology; the more severe an individual’s problem gambling symptoms, the more they typically spend on loot boxes^10–14^. This consistent positive association has been confirmed by a meta-analysis showing a robust association between problem gambling symptoms and loot box spending, and also between excessive gaming and loot box spending^14^. Taken together, these findings suggest a clear overlap in symptoms between behavioural addictions and loot box spending behaviours.

Some have argued that loot boxes have benefits. Such arguments are almost entirely ideological in nature and often lacking empirical evidence. For instance, some have argued that loot boxes are enjoyed by gamers. This argument is irrelevant; whether people enjoy an activity is not diagnostic of whether it is gambling. Many individuals also enjoy gambling, and this does not mitigate its potential harms for some gamblers. Moreover, the argument is not even true. Although evidence suggests a very small positive association between positive mood and loot box purchasing, this is balanced by an equivalently sized positive association with negative mood (i.e., more loot box spending is associated with higher negative mood^10^), suggesting overall mood is not related to loot box spending. Players who spend more on loot boxes have also been shown to have greater levels of post-purchase consumer regret, an emotion antithetical to enjoyment^15^, and some evidence suggests that greater purchasing might be associated with increased psychological distress^10,16,17^. Others have argued that loot boxes reduce the cost of games to players who do not purchase them, therefore providing overall community benefit^18^. There is no empirical evidence to support this claim. Indeed, this argument is predicated on the notion that those who purchase loot boxes are wealthy gamers with much disposable income and whose purchases support the community (i.e., “Whales”)^19^. This has been disproven in two separate analyses which show that income plays little-to-no role in loot box spending, and indeed it is consistently individuals with increased risk of gambling harm who consistently spend more on the mechanism across every income bracket^19,20^. Thus, far from socialising the benefits of loot boxes, paid for by the wealthy few, instead any such benefits received by non-paying gamers are disproportionately at the financial expense of gamers at increased risk of gambling harms. Moreover, the argument mirrors a long-standing myth in gambling research—that lotteries, with their revenue often directed to public spending, are the least harmful form of gambling. This myth has resulted in a dearth of research into lotteries, but recent research has shown the thesis to be untrue, suggesting that lotteries are associated with harm in a substantial subpopulation of users^21–23^. Thus, the argument that loot boxes are beneficial to many gamers must be weighed against the fact that disproportionately, revenue is drawn from vulnerable gamers at risk of gambling harm and not wealthy gamers and that, like National Lotteries, harm minimisation efforts to protect these vulnerable gamers are needed. To achieve this, it is essential to understand which players are at risk of increased spending on loot boxes, a question tackled by the present study.

Risky engagement with loot boxes and non-randomised microtransactions represents one facet of the literature on potentially harmful effects of video games on their users. In addition to the potential for risky engagement (e.g., overspending) with loot boxes and non-randomised microtransactions, gaming also has the potential to be engaged in excessively and contribute to harms for gamers^12^. Although there remains discussion in the literature about the operationalisation and symptoms associated with gaming disorder per se^24^, there is broad agreement that at least some who play video games do so to excess, and may experience difficulty curbing their gaming behaviours^25^. Thus, there is a degree of heterogeneity in the terminology used to describe excessive gaming, in the way this construct is operationalised by researchers. Our aim is not to disentangle this literature. Rather, we focus on the basic idea that gamers may engage in gameplay to excess, and this may result in negative outcomes. We use the term "problematic gaming" to encompass such negative outcomes.

When considering the likelihood that gamers might experience adverse outcomes associated with either in-game monetisation systems or excessive gameplay, a consideration of individual difference variables is likely to be useful. Previous research has demonstrated that various individual difference factors are associated with increased problematic gaming behaviours. For example, problematic gaming behaviours are positively associated with neuroticism and negatively associated with conscientiousness and openness to experience^26^. Further, Garrett et al.^27^ report that both positive urgency and sensation seeking (theorised elements of impulsivity) are positively associated with loot box spending. Psychological distress (anxiety, depression, and stress) is also positively associated with both problematic gaming behaviours^28^ and loot box expenditure^10,16^. Recent evidence also suggests a positive association between obsessive compulsive disorder (OCD) symptomatology and loot box spending^15^. Interested researchers have also begun exploring the relationships between neurodevelopmental differences, such as autism and ADHD, with use of technology, including internet addiction, video gaming behaviours, and engagement with in-game features^29–31^.

The American Psychiatric Association defines autism (‘autism spectrum disorder’), as a developmental disorder characterised by deficits in social communication and interactions, in combination with restrictive or repetitive patterns of thoughts, behaviour and interests^32^. In line with calls to change the language used to describe autistic people and their experiences, and language suggestions put forth by Monk et al.^33^, we will use identity-first language, and refer to “experiences/characteristics” instead of “symptoms.” However, when referring to specific psychometric scales or diagnostic terms, the language used in the original content matter will be retained.

Previous research has hypothesised that people with characteristics and experiences of autism may be particularly vulnerable to maladaptive video gaming, posited to be due to gaming becoming a specialised interest (termed ‘restricted interest’ within the DSM-5)^34^. However, a 2022 meta-analysis reported that high video game use within young, autistic male cohorts can be better explained by an addictions framework, with weaker evidence to suggest this relationship can be explained by specialised interests^35^. Furthermore, some have theorised that experiences common to neurodiverse populations may influence gaming behaviour, such as ‘hyperfocus’. Hyperfocus refers to a phenomenon whereby a person may become intensely focused on a task for a prolonged period of time, during which they may struggle to attune to external stimuli^36^. In fact, a recent study reported that higher hyperfocus scores strengthened the relationship between scores of ADHD and internet addiction^36^. While both specialised interests and hyperfocus represent interesting avenues of research in the neurodiversity-gaming literature, to foreshadow, our results do not provide support for these hypotheses.

There is a paucity of research investigating how in-game microtransaction spending is experienced by people on the autism spectrum. However, there are known relationships between parallel factors, such as the link between problematic gaming behaviours and autism. For example, several papers and two systematic reviews report positive associations between autism/autistic characteristics and experiences and problematic video game use in child, adolescent, and young adult cohorts^34,37–42^. However, the systematic reviews also list several limitations within the autism-problematic gaming literature, including methodological differences in age cohorts examined and operationalisations of problematic or disordered gaming status and/or behaviours^41,42^. Nonetheless, there is clear evidence suggesting that autistic gamers may be at higher risk of problematic engagement with video games, although as we explain below, the relationship between autistic experiences and traits and gambling is less clear. The justification to investigate microtransaction expenditure amongst autistic gamers is further strengthened when considering evidence that demonstrates a consistent association between problematic gaming and microtransaction expenditure in general populations^10,12–14^. With autism linked to excessive gameplay, and microtransaction expenditure associated with excessive gameplay, there is reason to expect that gamers on the autism spectrum may be likely to spend more on microtransactions than allistic (non-autistic) individuals.

Furthermore, related work on the relationship between problematic gambling symptomatology and autism also suggests there is merit in exploring the relationship between autism and in-game spending. Chamberlain et al.’s systematic review^43^ found no clear or consistent pattern of performance on gambling and decision-making tasks for autistic people compared to allistic (non-autistic) people. However, the authors emphasized the lack of research in the autism-gambling area^43^. While the autism-gambling relationship requires further exploration, the possible link between autistic characteristics/experiences and gambling provides some justification for examining how in-game spending—particularly on gambling-like microtransactions—is experienced by gamers on the autism spectrum. This justification is further strengthened when considering the consistent positive associations between problematic gambling and loot box expenditure^10–14^. With autism/autistic characteristics demonstrating some links to gambling, and gambling positively associated with loot box expenditure, autistic characteristics/experiences may also be associated with expenditure on loot boxes and non-randomised microtransactions. Better understanding how neurodevelopmental differences such as autism relate to video game use and in-game spending is important. This relationship is not well understood, meaning the potential risks for those with neurodevelopmental differences remain unclear.

### The present research

Based upon the above literature, we predicted that autistic characteristics and experiences would be positively associated with both in-game expenditure, and measures of risky gaming, gambling, and loot box engagement.

We had two categories of hypotheses. First, we tested correlations between measures of autistic characteristics and experiences, risky loot box engagement, problematic gambling, problematic gaming behaviours, and both loot box and non-randomised microtransaction expenditure. Second, we investigated the influence of autistic characteristics and experiences on the relationships between risky loot box engagement, problematic gambling, problematic gaming behaviours and both loot box and non-randomised microtransaction expenditure. Problematic gaming behaviours were measured by the Internet Gaming Disorder Checklist (IGD)^44^, problematic gambling by the Problematic Gambling Severity Index (PGSI)^45^, risky loot box engagement by the Risky Loot Box Index^46^, and autistic characteristics and experiences by the Ritvo Autism and Asperger Diagnostic Scale (RAADS-14)^47^. A description of the scales used can be found in the “Measures” section.

Therefore, we pre-registered the following hypotheses (https://osf.io/fkmaq/):

1. We predicted there would be a positive correlation between RAADS-14 score and the amount of money participants report spending on (a) randomised; and (b) non-randomised in-game purchases.
2. We predicted there would be a positive correlation between participants’ IGD score and the amount of money participants report spending on (a) randomised; and (b) non-randomised in-game purchases.
3. There would be a positive correlation between participants’ PGSI score and the amount of money participants report spending on (a) randomised; and (b) non-randomised in-game purchases.
4. There would be a positive correlation between participants’ Risky Loot Box Index score and the amount of money participants report spending on (a) randomised; and (b) non-randomised in-game purchases.
5. There would be a positive correlation between RAADS-14 scores and Risky Loot Box Index scores.
6. There would be a positive correlation between RAADS-14 scores and PGSI scores.
7. There would be a positive correlation between PGSI scores and IGD scores.
8. There would be a positive correlation between IGD scores and Risky Loot Box Index scores.
9. There would be a positive correlation between PGSI scores and Risky Loot Box Index scores.
10. We predicted that RAADS-14 scores will moderate the relationship between IGD scores and reported spending. The slope of the regression line for the association between IGD scores and reported in-game spending will be greater for participants with high (+ 1SD), compared to low (− 1SD) RAADS-14 scores.
11. RAADS-14 scores would moderate the relationship between PGSI scores and reported spending. The slope of the regression line for the association between PGSI scores and reported in-game spending will be greater for participants with high (+ 1SD), compared to low (−1SD) RAADS-14 scores.
12. RAADS-14 scores would moderate the relationship between Risky Loot Box Index scores and reported spending. The slope of the regression line for the association between Risky Loot Box Index scores and reported in-game spending will be greater for participants with high (+ 1SD), compared to low (− 1SD) RAADS-14 scores.

### Exploratory hypotheses

We also pre-registered one exploratory hypothesis about the association between RAADS-14 and IGD score. At the time of pre-registration, we lacked a compelling theoretical basis for predicting a direction of this association. As noted above, there are some limitations within the autism-problematic gaming literature, one being that research is often conducted only with clinical samples. In fact, in their systematic review, Murray et al.^42^ found seven studies on autism and gaming disorder eligible for analysis, with only two of these being conducted with non-clinical samples. This is an important gap within the literature, as over-representation of clinically diagnosed autistic participants in research may impair the generalisability of study findings to the broader population of individuals with autistic characteristics and experiences who do not reach clinical thresholds^48^. As such, our study asked a non-clinical sample for their agreement with characteristics or experiences consistent with autism, and we did not screen for autism diagnosis itself. However, based upon the above evidence demonstrating relationships between autism, video gaming and gambling, we believed that the relationship between autistic characteristics/experiences and problematic gaming warranted further investigation.

## Method

### Ethics

Ethics approval was obtained for human data collection, granted by the University of Tasmania’s Human Research Ethics Committee (reference number: H0025046). The research was performed in accordance with relevant guidelines and regulations, and participants gave informed consent to participate.

### Pre-registration

This study was pre-registered on the Open Science Framework. Our planned hypotheses and methods (including data collection, analyses, and exclusion and inference criteria) can be accessed at: https://osf.io/fkmaq/.

### A-priori power analysis

This study used a combination of frequentist and Bayesian analyses. Our a-priori power calculation based on frequentist analyses was used as a guide for determining minimum required sample size. We conducted the power analysis in G*Power, with pre-set power (1 − β = 0.95), a medium effect size (Cohen’s *d* = 0.5) and with statistical significance set at 0.05. Results indicated a minimum sample size of 184 participants. Because we were uncertain about the size of the effect we might expect to find, we sought to recruit 1200 participants to allow for us to be confident in effect sizes as small as *r* = 0.10.

### Design

We based our design on past work assessing the relationship between loot boxes and problematic gaming and problematic gambling symptomatology^10,14,46,49–52^. This is a cross-sectional between-subjects correlational design. Concordant with established methodology^14^, we created an online survey containing dependent measures of in-game expenditure (self-reported) and risky engagement with loot boxes (measured by the Risky Loot Box Index), and independent measures assessing problematic gaming behaviours (measured by the IGD), problem gambling symptoms (measured by the PGSI), and autistic characteristics and experiences (measured by the RAADS-14). We also collected basic demographic information, such as age, gender, and weekly income.

### Participants

Participants were recruited through Prolific Academic, with the survey distributed only to Prolific users from Australia, Aotearoa, and the US. Inclusion criteria also required all participants to be aged 18 years or older. A total of 1200 participants were recruited, however a data collection error with the survey collection platform led to 9 participant responses being lost, resulting in 1191 participants.

### Exclusions

Exclusion criteria were adapted from Drummond et al.^10^, who measured a similar cohort and variables to the present study. Using a pairwise exclusion method, participant data were excluded according to the following pre-registered exclusion criteria:

1. Participants who responded ‘yes’ to the statement “I once owned a three headed dog”.
2. Participants who responded with anything other than ‘3’ for the statement “please respond 3 to this question”.
3. Any participant who reported that they have spent more than $1000 on loot boxes in the past month.
4. Any participant who failed to respond to 75% of the questions from a scale, would be excluded from analyses using that scale.

First, a total of 12 participants were excluded for failing the deception and attention checks. Secondly, one participant was excluded for reporting loot box expenditure exceeding $1000 over the past month. Following these exclusions, the final sample consisted of 1178 participants. The fourth exclusion criteria stipulated that any participant who did not respond to 75% of the questions from a scale, would be excluded from analyses using that scale. This resulted in three participants being excluded from analyses using the PGSI (sample = 1175), and four participants were excluded from analyses using the Risky Loot Box Index (sample = 1174). For participants who did not answer 100% of the items from any scale but answered more than 75% (n = 44), we assigned a score of 0 to the missing values (in the relevant scales) and summed the total of the scale items. For completeness, analyses were conducted both with and without these 44 participants included. No meaningful difference of results was found when excluding these participants. Lastly, a further two participants did not elect a country of residence, and therefore their expenditure in US currency was not able to be calculated, resulting in these two cases being excluded from analyses involving expenditure on games, loot boxes or non-randomised microtransactions. Both data sets, with and without the excluded participants and their associated analyses, can be found at: https://osf.io/fkmaq/.

## Measures

### Loot box expenditure

The first critical spend variable measured how much money was spent on loot boxes (i.e., randomised in-game items). As per Drummond et al.^10^, participants were asked to report how much money (in their local currency) they had spent on loot boxes in the past month. Australian and Aotearoa participant loot box spend data was converted into US dollars using Google’s conversion rates from the 28th of April 2023 (the day of data collection). Recorded spend data from Australian participants was multiplied by 0.6616 and Aotearoa spend data was multiplied by 0.6182. Note that in response to an anonymous reviewer comment we also reanalysed our data using loot box expenditure as a categorical variable, and also excluding individuals who did not purchase loot boxes. These analyses yielded qualitatively similar results and as such we report the pre-registered analyses herein. Interested readers can obtain the open access dataset for reanalysis from the OSF link listed in the data availability statement.

### Non-randomised microtransaction expenditure

The second critical spend variable measured how much money was spent on non-randomised in-game items. Participants were asked to report how much money (in their local currency) they had spent on non-randomised in-game items in the past month. Spend data were converted to USD as described above.

### Problematic gaming

Problematic gaming was measured by the Internet Gaming Disorder Checklist (IGD)^44^. The IGD checklist is a nine-item scale based upon the proposed diagnostic criteria for internet gaming disorder. Participants answer the nine items on a four-point Likert scale, with responses ranging from 1 (not at all true) to 4 (very true). Example statements include: “I have lost interest in other hobbies or entertainment in order to play games” and “I feel irritable, anxious or sad when I am unable to game.” Summing scores from all nine items produces a total IGD score ranging between 9 and 36, with higher scores indicating greater problematic gaming behaviours. Internal reliability of the IGD was high (α = 0.847).

### Problem gambling symptoms

Problem gambling symptoms were measured using the Problem Gambling Severity Index (PGSI)^45^. The PGSI consists of nine items and asks participants to indicate over the past 12 months how frequently (0, never—3, almost always) they experienced problems caused by their gambling behaviours. Example scale items include “have you bet more than you could really afford to lose?” and “has gambling caused you any health problems, including stress or anxiety?” Summed PGSI scores range from 0 to 27, with higher scores indicating greater problem gambling symptoms. Internal reliability of the PGSI was high (α = 0.950).

### Risky loot box engagement

The Risky Loot Box Index^46^ was used to measure risky cognitions and behaviours regarding loot box expenditure. The Risky Loot Box Index asks participants to rate their agreement (1, strongly disagree—7, strongly agree) with five statements regarding their engagement with loot boxes. Example scale items include: “the thrill of opening loot boxes has encouraged me to buy more” and “I frequently play games longer than I intend to, so I can earn loot boxes.” Summed scores from the five items range between 5 and 35, with higher scores indicating riskier engagement with loot boxes. Internal reliability of the Risky Loot Box Index was high (α = 0.910).

### Autistic characteristics and experiences

Autistic characteristics and experiences were measured using the Ritvo Autism and Asperger Diagnostic Scale (RAADS-14)^47^, a self-report questionnaire designed to quickly screen adults for ASD symptomatology and for possible further ASD assessment. The RAADS-14 consists of 14 statements, each measuring various life experiences or personality characteristics (i.e., symptoms) that are consistent with ASD (e.g., “it is difficult for me to understand how other people are feeling when we are talking”). Participants are asked to choose one of four alternative responses, indicating the duration of the autistic trait over the lifespan. Response options (scored 0–3, apart from one reverse scored item) include: 0—never true, 1—true only when I was younger than 16, 2—true only now, and 3—true now and when I was young. Summed RAADS-14 scores range between 0 and 42, with a recommended cut-off score of 14. During the development of the RAADS-14, validity testing with the cut-off score of 14 indicated a sensitivity of 0.97, and a specificity of 0.95 in non-psychiatric controls. The RAADS-14 was selected for the present research based on its reported psychometric strengths for a short-form scale. This scale was used in the present research as a measure of autistic characteristics and experiences, with higher scores representing higher concurrence with possible autistic traits. Internal reliability of the RAADS-14 was high (α = 0.874).

### Results analysis plan

Initially, we inspected variables for high degrees of skewness or kurtosis (> 2). Four variables were found to have high skewness and kurtosis scores, including video game expenditure, loot box expenditure, non-randomised microtransaction expenditure and overall PGSI score. Following pre-registered transformation protocols, we rank order transformed each of these variables for subsequent analyses.

We used Bayesian correlations to assess the relationships between our critical spend variables—reported loot box expenditure and non-randomised microtransaction expenditure—and overall scores for the IGD, Risky Loot Box Index, PGSI (rank ordered) and RAADS-14 scales (H1–H9, exploratory hypothesis). Where feasible (given statistical package limitations), Bayesian analyses were selected in favour of null hypothesis significance testing (NHST). One benefit of Bayesian analyses over NHST methods is that it can quantify evidence for the null hypothesis. In other words, NHST methods can only discern an “absence of evidence”, whereas Bayesian analyses can quantify “evidence of absence”^53^. We interpreted Bayes factors according to the criteria outlined by Lee and Wagenmakers^54^, which stipulate that Bayes factors above 1 indicate evidence for the alternative hypothesis, and Bayes factors below 1 indicate evidence for the null hypothesis. For the alternative hypothesis, Bayes factors between 1 and 3 is considered anecdotal evidence, 3–10 is moderate evidence, 10–30 is strong evidence, 30–100 is very strong evidence, and 101+ is extreme evidence. Conversely, for the null hypothesis, Bayes factors between 0.33–1 is considered anecdotal evidence, 0.10–0.33 is moderate evidence, 0.03–0.10 is strong evidence, 0.01–0.03 is very strong evidence, and less than 0.01 is extreme evidence. We used Pearson’s *r* as a measure of effect size, with *r* values < 0.1 representing a negligible effect, between 0.1 and 0.3 a weak effect, between 0.29 and 0.49 a moderate effect, and > 0.5 representing a strong effect^55^. Moderation analyses were then used to examine the effect of RAADS-14 score on the relationships between spend variables and overall scores on the IGD, Risky Loot Box Index and PGSI (rank ordered) (H10–H12). The moderation analyses required a frequentist approach due to the statistical package not offering a Bayesian option for moderations. All analyses were conducted on jamovi using the Bayesian Methods (jsq) and MedMod packages, with bootstrapping of 1000 samples^56–60^.

## Results

### Participant demographics and gaming behaviours

The final sample consisted of 1178 participants, including 907 (77.1%) from the US, 235 (20%) from Australia, and 34 (2.9%) from Aotearoa (two did not specify country of residence). The average age of respondents was 36.7 years (SD = 12.4), with 702 (59.6%) participants identifying as male, 441 (37.4%) as female, and 30 (2.5%) as non-binary. Four (0.3%) participants preferred not to disclose their gender, and 1 (0.1%) participant identified as ‘other’. Further demographic statistics are included within the supplementary materials**.**

A vast majority of participants indicated they had played a video game in the past month (95.2%), with 38.9% of respondents (n = 458) reporting they gamed almost every day. On average, participants reported spending US$39.48 (SD = 98.50) on video games, US$16.58 (SD = 94.25) on non-randomised microtransactions, and US$12.41 (SD = 51.02) on loot boxes in the past month. The average expenditure for each spend variable appears to be relatively small, which is likely due to both wide ranges for reported expenditure across each spend variable, and a high proportion of non-spenders among the sample. A majority of the participants reported no expenditure on video games (51.9%), loot boxes (77%) and non-randomised microtransactions (67.7%) in the past month.

### Bayesian correlations

Tables 1 and 2 show the relationships between our critical spend variables, loot box and non-randomised microtransaction expenditure, with autistic characteristics and experiences (RAADS-14 score), problematic gaming behaviours (IGD scores), gambling symptomatology (PGSI scores), and Risky Loot Box Index scores. Counter to expectations, the correlation between RAADS-14 scores and loot box expenditure (H1a) yielded very strong evidence for the null hypothesis (Table 1). Similarly, there was anecdotal evidence for the null when correlating RAADS-14 scores and non-randomised microtransaction expenditure (H1b, Table 1). Overall, this suggests that there is no relationship between autistic characteristics/experiences and non-randomised microtransaction or loot box expenditure. Conversely, there was extreme evidence (BF_10_ > 100) for positive correlations between IGD, PGSI, and Risky Loot Box Index scores and loot box and non-randomised microtransaction expenditure (H2–H4), with correlation coefficients indicating weak-to-strong associations (Table 1).

**Table 1:** Loot boxes and non-randomised microtransactions-Bayesian correlations.

	Pearson’s r	95% CI	BF_10_
Loot boxes			
RAADS-14	− 0.031	0.00, 0.05	0.02
IGD	0.264	0.21, 0.32	1.67138 × 10^17^
PGSI	0.341	0.29, 0.39	1.89 × 10^30^
Risky Loot Box Index	0.545	0.50, 0.58	1.25 × 10^88^
Non-randomised microtransactions			
RAADS-14	0.060	0.01, 0.12	0.58
IGD	0.317	0.26 0.37	7.11 × 10^25^
PGSI	0.245	0.19, 0.30	3.61375 × 10^14^
Risky Loot Box Index	0.363	0.31, 0.41	4.66 × 10^34^

Relationships between loot box and non-randomised microtransaction expenditure with autistic characteristics/experiences (RAADS-14), problematic gaming (IGD), problem gambling symptomatology (PGSI), and risky engagement with loot boxes (Risky Loot Box Index).

**Table 2:** Bayesian correlations between scales.

	IGD		PGSI		Risky Loot Box Index	
	Pearson’s r [95% CI]	BF_10_	Pearson’s r [95% CI]	BF_10_	Pearson’s r [95% CI]	BF_10_
RAADS-14	0.291 [0.24, 0.34]	2.81 × 10^21^	0.136 [0.08, 0.19]	4347.06	0.093 [0.04, 0.15]	11.93
IGD	–	–	0.383 [0.33, 0.43]	1.98 × 10^39^	0.525 [0.48, 0.56]	7.38 × 10^80^
PGSI			–	–	0.448 [0.40, 0.49]	4.96 × 10^55^
Risky Loot Box Index					–	–

Pearson’s correlations and Bayes factors between autistic characteristics/experiences (RAADS-14), problematic gaming (IGD), problem gambling symptomatology (PGSI) and risky engagement with loot boxes (Risky Loot Box Index).

Relationships between the gaming, gambling, risky loot box engagement and autistic characteristics/experiences variables are shown in Table 2. RAADS-14 scores and Risky Loot Box Index scores (H5) showed a negligible positive relationship, while RAADS-14 and PGSI (H6), and RAADS-14 and IGD (exploratory hypothesis) demonstrated weak positive relationships. The strongest associations found were between our gaming, gambling, and risky loot box variables, with moderate associations between PGSI and IGD scores, and PGSI and Risky Loot Box Index scores. Lastly, a strong, positive association was found between Risky Loot Box Index and IGD scores (H7–H9).

### Moderation analyses

We ran moderation analyses to investigate the impact of RAADS-14 on the relationships between our scale variables and critical spend variables (H10–H12). We expected that RAADS-14 score would moderate the relationship between microtransaction expenditure and PGSI score, IGD score, and Risky Loot Box Index score. Two moderation analyses were run per hypothesis—one looking at loot box expenditure and the other looking at non-randomised microtransaction expenditure. Across analyses, three consistent patterns emerged. First, there were significant main effects of RAADS-14 scores on loot box expenditure in all moderation analyses (IGD, PGSI, Risky Loot Box Index). In each of these analyses, results demonstrated negative associations, such that higher levels of autistic characteristics and experiences was associated with lower loot box expenditure. Second, however, RAADS-14 scores were not significantly associated with non-randomised microtransaction spending in any of the moderation analyses. Finally, there were no significant interactions in any moderation analysis, providing no evidence that RAADS-14 scores moderated the consistently significant and positive associations between PGSI, IGD, and Risky Loot Box Index scores and spending on non-randomised microtransactions and loot boxes. All moderation results can be seen in Table 3. Moderation results are presented with frequentist statistics due to statistical limitations of the analysis program used.

**Table 3:** Moderation estimates.

Predictor variables			95% CI			
	Estimate	SE	Lower	Upper	Z	*p*
Loot box spend as the dependent variable, RAADS-14 score as the moderator						
IGD						
Direct effect—IGD	14.894	1.556	11.78	18.09	9.571	< 0.001
Direct effect—RAADS-14	− 2.821	0.677	− 4.09	− 1.47	− 4.169	< 0.001
Interaction—IGD*RAADS-14	− 0.161	0.143	− 0.44	0.11	− 1.126	0.260
PGSI						
Direct effect—PGSI	0.303	0.029	0.24	0.36	10.616	< 0.001
Direct effect—RAADS-14	− 1.877	0.665	− 3.16	− 0.57	− 2.821	0.005
Interaction—PGSI*RAADS-14	− 0.000	0.003	− 0.01	0.00	− 0.162	0.872
Risky Loot Box Index						
Direct effect—Risky Loot Box Index	16.249	0.766	14.71	17.70	21.226	< 0.001
Direct effect—RAADS-14	− 2.025	0.589	− 3.16	− 1.01	− 3.438	< 0.001
Interaction—Risky Loot Box Index*RAADS-14	− 0.132	0.071	− 0.29	− 0.01	− 1.852	0.064
Non-randomised microtransaction spend as the dependent variable, RAADS-14 score as the moderator						
IGD						
Direct effect—IGD	18.372	1.631	15.12	21.63	11.262	< 0.001
Direct effect—RAADS-14	− 0.943	0.785	− 2.43	0.71	− 1.202	0.229
Interaction—IGD*RAADS-14	− 0.137	0.162	− 0.45	0.20	− 0.846	0.398
PGSI						
Direct effect—PGSI	0.231	0.030	0.17	0.29	7.803	< 0.001
Direct effect—RAADS-14	0.805	0.762	− 0.78	2.34	1.057	0.291
Interaction—PGSI*RAADS-14	0.001	0.003	− 0.00	0.01	0.499	0.618
Risky Loot Box Index						
Direct effect—Risky Loot Box Index	11.888	0.946	9.89	13.71	12.562	< 0.001
Direct effect—RAADS-14	0.774	0.748	− 0.65	2.29	1.034	0.301
Interaction—Risky Loot Box Index*RAADS-14	− 0.007	0.084	− 0.17	0.14	− 0.085	0.932

## Discussion

We investigated the relationships between autistic characteristics/experiences, problematic gaming, problematic gambling, risky loot box use, and non-randomised microtransaction and loot box spending. Overall, autistic characteristics and experiences were not associated with increased loot box and non-randomised microtransaction spending, but were positively associated with gambling symptomatology, problematic gaming behaviours, and risky loot box use. Counter to predictions, when included with problem gambling, excessive gaming symptoms, or risky loot box use, autistic characteristics and experiences were associated with a small reduction in spending on loot boxes. For ease of discussion, the correlational results will be discussed in terms of replicated results and novel results.

In terms of replicated results, problematic gaming, problematic gambling, and risky loot box use (H2–H4), were all positively associated with spending on both loot boxes and non-randomised microtransactions. Furthermore, participants with higher PGSI scores had higher problematic gaming scores (H7) and higher risky loot box use scores (H9), and participants with higher problematic gaming scores had higher risky loot box use (H8). These results replicate prior research demonstrating positive associations between problematic gaming behaviours and problem gambling symptomatology^10,46^, problematic gaming behaviours and risky loot box use^10,46^, and problem gambling symptomatology, problematic gaming behaviours, and risky loot box use with increased loot box spend^10–14,46^.

The remaining correlational (H1, H5, H6, exploratory hypothesis) and moderation (H10–H12) hypotheses all concern our novel research results. These remaining hypotheses predicted positive associations between characteristics and experiences consistent with autism, in-game expenditure variables, and our other scale variables of interest. Participants with higher reports of autistic characteristics/experiences had higher levels of self-reported problematic gaming, problematic gambling, and risky loot box use, albeit it with negligible-to-weak strength of associations (H5, H6, exploratory hypothesis). However, our predictions regarding a positive association between autistic characteristics/experiences and in-game expenditure were not supported. The results indicated very strong evidence for a null relationship between autistic characteristics/experiences and loot box spending (H1a), and anecdotal evidence for a null relationship between autistic characteristics/experiences and non-randomised microtransaction spend (H1b). These results are interesting, suggesting gamers with autistic characteristics experience higher levels of problematic gaming, problematic gambling, and risky loot box use, but do not report higher levels of spending on in-game items. This is curious, given our replication of previous findings demonstrating that in-game expenditure is related to problematic gaming behaviours, problematic gambling symptomatology, and risky loot box use. We return to this point after considering the results of the moderation analyses.

We also predicted that higher levels of autistic characteristics/experiences would compound established associations between loot boxes and non-randomised microtransaction spending and (a) problem gaming behaviours, (b) problematic gambling symptomatology, and (c) risky loot box engagement (H10–H12). However, in all cases, we found no statistical evidence supporting these hypotheses. The moderation analyses did, however, provide support for our correlational results, revealing a possible difference between loot box expenditure and non-randomised microtransaction expenditure. Results consistently showed that when controlling for IGD, PGSI, or Risky Loot Box Index scores, there was no evidence of a relationship between autistic characteristics/experiences and non-randomised microtransaction spending, but that there was evidence for a small negative relationship between autistic characteristics/experiences and loot box spending, such that higher levels of autistic characteristics/experiences were actually associated with *lower* spending on loot boxes. Although we cannot provide definitive explanations for these findings based on the current data, we can offer some potential theoretical explanations and identify possible avenues for future research.

Within the autism-gaming literature, it has been hypothesised that individuals on the autism spectrum may be more vulnerable to excessive gameplay^34^. Indeed, our results support the notion that gamers with higher levels of autistic characteristics/experiences have higher levels of problematic gaming. This finding has similarly been reported in several clinical cohort studies^34,37–40^. However, before the present study, this had not been investigated in broader, non-clinical samples of adults with varying levels of autistic characteristics. Interestingly, this effect did not extend to in-game expenditure on either loot boxes or non-randomised microtransactions. Thus, our findings raise an interesting question: why are gamers higher in autistic characteristics/experiences reporting higher levels of problematic gaming, gambling, and loot box engagement, but are not spending greater amounts of money on in-game purchases?

One possible explanation draws upon research in reasoning and decision making. De Martino et al.^61^ found that individuals with autistic characteristics are less susceptible to framing effects, where decisions are based more on the presentation of information than the utility of the underlying options. Further to this, recent work suggests that compared to allistic (non-autistic) individuals, autistic individuals tend to process information more deliberately (slowly, system 2 thinking) rather than intuitively (quick, system 1 thinking)^62,63^. Theoretically, this more deliberative thinking style may allow autistic gamers to better consider the utility/worth of loot box purchases and avoid impulsive purchasing^27^. However, this explanation does not account for our finding that, compared to allistic (non-autistic) gamers, gamers with autistic characteristics also experience higher problem gambling symptomatology on average. Perhaps autistic gamers, being more likely to engage in slower, more deliberate decision-making processes, may consider the utility of loot boxes to be lower than the more tangible financial reward that gambling may yield.

Alternatively, perhaps loot boxes are particularly aversive to gamers with autistic characteristics. Loot boxes and more broadly, microtransactions, are commonly disliked within the gaming community. One prevailing grievance with these monetisation systems is the potential for unfair gameplay^64^. Essentially, those who can afford non-randomised microtransactions and loot boxes can gain access to in-game content that may increase in-game performance (i.e., in competitive multi-player environments) or experience^64^. Parallel to this, research has demonstrated that individuals with ADHD are higher in justice sensitivity; the tendency to perceive injustices more often and with more intense cognitive, emotional, and behavioural reactions^65,66^. Although this effect has not yet been investigated in autistic populations, anecdotal evidence suggests those with autistic characteristics and experiences may be similarly high in justice sensitivity^67–69^. Furthermore, there is substantial evidence demonstrating considerable overlap between autism and ADHD, including shared biological, behavioural, and cognitive features^70–72^. Therefore, it is tenable that autistic gamers may be particularly averse to the perceived unfairness of loot boxes. This possible explanation can be strengthened by considering traits and experiences that may be shared by autistic and ADHD gamers, such as ‘cognitive rigidity’. Cognitive rigidity refers to difficulties associated with ‘cognitive flexibility’, which is the ability to switch ways of thinking or behaving when new information or demands are presented^73^. Townes et al.’s^72^ recent meta-analysis found that both ADHD and autistic groups performed significantly worse than non-autistic groups on measures of cognitive flexibility, and did not differ significantly from each other. Although the mechanisms underlying cognitive flexibility/rigidity in autism and ADHD are not yet known as distinct or shared^72^, a more inflexible thinking style may result in neurodivergent gamers feeling resistant to engaging in randomised reward systems that both (a) yield unknown benefits and (b) can provide gamers with greater financial means ‘unfair’ in-game advantages. Furthermore, cognitive rigidity may strengthen any pre-existing beliefs around the fairness (or lack thereof) of loot boxes. We posit that given the overlap between autism and ADHD (including the shared trait of cognitive rigidity), and evidence showing heightened justice sensitivity in individuals with ADHD, perhaps gamers with autistic characteristics are similarly sensitive to perceived injustices. Thus, gamers higher in justice sensitivity may find loot boxes particularly unfair—and thus avoid purchasing these in-game features. Although our data do not allow us to draw firm conclusions about these explanations, these potential explanations represent possible avenues for future research into the autism-gaming field.

That autistic characteristics/experiences were positively associated with problematic gambling is particularly noteworthy. Although our findings support those of Grant et al.^74^, who similarly found a positive association between autistic characteristics/experiences and gambling, a recent meta-analysis and systematic review yielded divergent conclusions. Zeif and Yechiam’s^75^ meta-analysis found no evidence for an association between autism and performance on the Iowa Gambling Task. Conversely, Chamberlain et al.^43^ reported mixed findings regarding autism and gambling across a limited number of studies. Furthermore, it is puzzling that our results indicate that those higher in autistic characteristics/experiences are higher in problematic gambling symptomatology, yet spend less on loot boxes. Previous research has revealed a relationship between problem gambling and loot box spending, whereby the more severe a participants’ problem gambling symptoms, the more they spend on loot boxes^10–13^, a relationship that has been further verified in a meta-analysis^14^. While the association between gambling and loot boxes is clear, the relationships between autism, gambling, and loot boxes appears more complex. These contradictory findings demonstrate the importance of further research to better understand how loot boxes and gambling are experienced by individuals with autistic characteristics.

This research is limited by some elements of the sampling process. We acknowledge that our participant sample is relatively homogenous in terms of culture, coming from Western countries. As such, our findings may not cross-culturally replicate. We suggest that further research should be conducted to explore whether these associations are present within other cultures, such as China, South Korea and Japan^76^. Additionally, participants were recruited from the general population and were not screened for diagnosed autism. Thus, we cannot differentiate between participants with diagnosed autism, participants with undiagnosed autism, and participants who may not be autistic but share similar characteristics with individuals on the autism spectrum. Further, although the RAADS-14 reports good discriminant validity in ADHD populations^47^, it is possible that the present study’s survey captured participants with ADHD. Although autism and ADHD are highly co-occurring and share many overlapping features^70–72^, the distinctions between these neurodevelopmental differences may result in dissimilar use of video games and microtransaction spending. With little research being conducted on the relationships between neurodiversity and microtransaction expenditure, it is unknown whether possible co-occurring ADHD may exacerbate or weaken our study’s findings. Furthermore, we present findings from a non-clinical sample. This itself does not represent a significant limitation, as surveying a non-clinical sample accommodates participants who may not have received a formal diagnosis of autism. However, it is unknown if our results would replicate within narrower samples (e.g., clinically diagnosed populations), or within autistic populations with greater sensory and communication support needs. We propose that future studies should control for clinical autism status and possible co-occurring neurodevelopmental differences such as ADHD, to elucidate their possible effects upon video gaming and spending behaviours.

We investigated the relationships between autistic characteristics/experiences, problematic gaming, problematic gambling, risky loot box use, and non-randomised microtransaction and loot box spending for participants from Australia, Aotearoa, and the United States. Our results support previous findings within the literature—that those who experience higher problematic gaming behaviours, greater problematic gambling symptomatology, and riskier engagement with loot boxes, spend more money on both randomised and non-randomised in-game items. Moreover, that people with higher levels of problematic gameplay have riskier engagement with loot boxes, and that people with greater problematic gambling symptomatology experience higher levels of problematic gaming behaviours, and experience riskier loot box engagement. Furthermore, we present new insights into video game use by a potentially vulnerable community. The results show that people with higher levels of autistic characteristics/experiences have greater problematic gaming behaviours, and problematic gambling symptomatology. However, as prevalence of autistic characteristics/experiences increase, that amount spent on loot boxes decreases. This suggests that vulnerability to increased spending on loot boxes is more aligned with addictive behaviours, while autistic characteristics may be a slight protective factor when these symptoms are taken into account. Additionally, this research lends to the academic and policy discussion regarding regulation of microtransactions in video games. Internationally, policymakers have begun legislating or exploring regulatory options for loot boxes and non-randomised microtransactions^77–79^. While our research does not indicate that autistic gamers are at a higher risk of loot box expenditure than allistic (non-autistic) gamers, our findings replicate previous findings that gamers higher in problematic gaming and problematic gambling are spending more on loot boxes. As such, this research may inform policymakers in their considerations of regulating loot boxes to minimise the risk associated with over-expenditure on microtransactions for gamers experiencing problematic gaming or gambling behaviours. As mounting literature demonstrates the similarities between loot boxes and more traditional forms of gambling, and the potential for loot box spending to predict migration to more conventional forms of gambling^80,81^, it becomes increasingly important to better understand which gamers may be at increased risk of financial harm.

## Supplementary information

The online version contains supplementary material available at: Correspondence and requests for materials should be addressed to J.D.S.

### Supplementary Information

Supplementary Tables.

## Data Availability

The datasets analysed during the current study, and copies of the exact analyses are available in the Open Science Framework (OSF) repository*:* https://osf.io/fkmaq/.
