# Supplementary material for: The associations between autistic characteristics and microtransaction spending
Source: Sci Rep. 2024 Jun 18;14:14068. doi: 10.1038/s41598-024-64812-z (PMC11189550; doi:10.1038/s41598-024-64812-z)
Supplement: Supplementary file 1 — Supplementary Tables. [file 41598_2024_64812_MOESM1_ESM.docx]

**The associations between autistic characteristics and microtransaction spending**

Tegan Charnock^1^, Aaron Drummond^1^, Lauren C. Hall^2^, & James D. Sauer^1*^

**Supplementary Material**

Table S1 shows a sociodemographic breakdown of participant features, including; age, gender, country of residence and household income.

| **Table S1.** Participant’s Sociodemographic Characteristics | |
| --- | --- |
| Variable |  |
| Age (*M, SD)* | 36.7 (12.36) |
| Gender (n, %) |  |
| *Male* | 702 (59.6%) |
| *Female* | 441 (37.4%) |
| *Non-binary* | 30 (2.5%) |
| *Prefer not to say* | 4 (0.3%) |
| *Other* | 1 (0.1%) |
| Country of Residence (n, %) |  |
| *Australia* | 235 (20%) |
| *United States* | 907 (77.1%) |
| *Aotearoa* | 34 (2.9%) |
| Weekly Household Income (n, %) |  |
| *Less than $350* | 131 (11.1%) |
| *$351-$700* | 210 (17.8%) |
| *$701-$1650* | 429 (36.4%) |
| *$1651-$3500* | 296 (25.1%) |
| *More than $3500* | 112 (9.5%) |

*Note: Participant responses to weekly household income was recorded in the individual participant’s local currency. ‘Weekly household income’ data is not reflective of currency conversion rates between Australia, The United States, and Aotearoa.*

Table S2 shows the gaming behaviours of participants, including time spent playing video games, and expenditure on video games and microtransaction features.

| **Table S2.** Participants Gaming Characteristics | |
| --- | --- |
| Variable |  |
| Video Game Expenditure (*M, SD)* | 39.48 (98.50) |
| Loot Box Expenditure (*M, SD)* | 12.41 (51.02) |
| Non-randomised Microtransaction Expenditure (*M, SD)* | 16.58 (94.25) |
| Video Game Play over the previous month (n, %) |  |
| *Yes* | 1121 (95.2%) |
| *No* | 57 (4.8%) |
| Has purchased a microtransaction (n, %) |  |
| *Yes* | 879 (74.7%) |
| *No* | 281 (23.9%) |
| *Unsure* | 17 (1.4%) |
| Has used a gift card to purchase in-game items (n, %) |  |
| *Yes* | 538 (45.7%) |
| *No* | 639 (54.3%) |
| Frequency of Video Gaming (n, %) |  |
| *Never* | 54 (4.6%) |
| *Sometimes* | 337 (28.6%) |
| *Most Days* | 329 (27.9%) |
| *Almost Every Day* | 458 (38.9%) |

*Note: Expenditure figures presented in USD.*
